# Notable influences of estrogen and sex-specific microenvironment in colorectal cancer revealed by single-cell transcriptome analysis

**DOI:** 10.7150/ijms.106133

**Published:** 2025-05-28

**Authors:** Yihui Zheng, Chaoxin Yang, Guozhong Xiao, Mingyuan Lei, Pengfei Qin, Huaxian Chen, Hongcheng Lin

**Affiliations:** 1 Department of General Surgery (Department of Coloproctology), The Sixth Affiliated Hospital, Sun Yat-sen University, Guangzhou 510655, China; 2 BGI Research, Chongqing 401329, China; 3 Guangdong Provincial Key Laboratory of Colorectal and Pelvic Floor Diseases, The Sixth Affiliated Hospital, Sun Yat-sen University, Guangzhou 510655, China; 4 Biomedical Innovation Center, The Sixth Affiliated Hospital, Sun Yat-sen University, Guangzhou 510655, China; 5 BGI Research, Shenzhen 518083, China

**Keywords:** Colorectal cancer, Sex, Estrogen, Single-cell RNA sequencing, Tumor microenvironment

## Abstract

**Background:** Colorectal cancer (CRC), the third most common malignancy worldwide, exhibits notable sex-specific prognostic differences, yet the underlying biological mechanisms remain poorly understood. **Methods:** In this study, we conducted single-cell sequencing on 32 CRC samples, followed by pathway enrichment analysis, cell-cell interaction analysis, and transcription factor analysis. The co-expression of GZMB and the transcription factor EOMES in CD8+ T cells was detected using multiplex immunohistochemistry. Western blot and TUNEL assays were employed to validate estrogen-induced apoptosis in CRC cell lines.** Results:** After quality control, we obtained a total of 167,437 cells across 9 cell types from all samples. Specifically, our analysis revealed sex-based variations in cellular composition, functionality, and intercellular interactions within CRC. Notably, female CRC samples exhibited significant positive correlation between estrogen signaling pathway activation and apoptotic activity, with validation through Western blot and TUNEL assays confirming estrogen-mediated apoptosis induction in CRC cell lines. The immune response was notably enhanced in female CRC, with CD8+ T cells showing increased expression of the EOMES gene regulatory network, thereby boosting T cell immunity. Moreover, B cells of female CRC demonstrated improved capabilities in antigen-presenting and MHC-I interactions with T cells. Additionally, Macro_CCL4 cells engaged in sex-specific TNF-TNFRSF1B crosstalk with CD8+ T cells, potentially leading to enhanced antitumor immunity in females. Conversely, CAF_MMP11 cells exhibiting a myofibroblastic CAF phenotype interacted with malignant epithelial cells via signaling pathways such as THBS, MK, and FN1, likely promoting CRC progression.** Conclusions:** Our research highlights the distinct immunological and hormonal responses in CRC by sex, which may explain the observed prognostic disparities. These findings may offer additional further biological insights for targeted therapies in CRC.

## Introduction

Colorectal cancer (CRC) ranks as the third most prevalent malignancy globally, characterized by notably high incidence and mortality rates 1. Epidemiological data indicate that both incidence and mortality rates of CRC are higher in men than in women 2. The 10-year cumulative fatality from CRC remains consistently elevated in males compared to females throughout the 50 - 75 age span 3. The observed disparity in survival rates between sexes underscores fundamental biological differences in CRC cancer pathogenesis and treatment responses.

Factors such as anatomical location, obesity, sex hormones, and gut microbiota may all influence the survival rates of colorectal cancer differently across sexes. Women exhibit a higher incidence of right-sided colon cancer compared to men, with right-sided tumors generally exhibiting a more aggressive phenotype than left-sided tumors 4, 5. Epidemiological studies indicate that obesity, which is strongly associated with men, is a significant risk factor for CRC^6^. Hormones such as estrogen, progesterone, and testosterone have been shown to exert modulatory effects on the immune system, influencing both cellular composition and the cytokine secretion profiles 7. Furthermore, estrogen also plays a protective role in colorectal cancer pathogenesis through modulating various signaling pathways, including the Wnt/β-catenin pathway 8. Additionally, research has demonstrated that male-biased gut microbiota metabolites promote the CRC progression via the glycerophospholipid metabolism pathway 9.

The tumor microenvironment (TME), which comprises the immediate surroundings of tumor cells, creating a complex milieu that includes blood vessels, tumor cells, immune cells, stromal cells, various signaling molecules, and extracellular matrix (ECM). This intricate environment is pivotal for the tumor cell survival and progressions. Sex-specific risk factors, particularly those related to hormonal influences, can markedly influence the composition and functionality of the TME. Such factors may modify the interactions and signaling pathways between various cellular components, leading to significant variations in clinical outcomes. Sex-specific disparities in immune cell composition and signaling pathways have been thoroughly investigated across various cancers. For instance, activated CD8+ T cells are more prevalent in females in cases of lung squamous cell carcinoma and stomach adenocarcinoma, whereas higher levels are observed in males in pheochromocytoma and paraganglioma, kidney renal papillary cell carcinoma, and liver hepatocellular carcinoma 10. In colorectal cancer, females exhibit a greater abundance of Th1 cells and cytotoxic T cells, suggesting a more robust immune response against tumor cells 11. Yang et al. reported that male CD8+ T cells demonstrate reduced effector and stem-like characteristics compared to their female counterparts 12. Furthermore, the Cancer Genome Atlas (TCGA) data analysis also reveals a higher relative abundance of CD4+ and CD8+ T cells in female CRC patients, indicating a potential immunological advantage 13. Estrogen is a key factor in sex -based disparities within the CRC tumor microenvironment, as it plays a crucial role in influencing the tumor microenvironment to inhibit tumor growth and progression. It is known to impede CRC progression via NRF2 pathways by promoting apoptosis, inhibiting the c-Myb protein, and downregulating the transcription of the anti-apoptotic protein Bcl-2 14. In the TME of CRC, estrogen may also curb tumor growth by reducing PD-L1 expression and increasing the M1 macrophage population 15.

Previous studies have highlighted the pivotal influence of sex-related disparities of the tumor microenvironment on survival outcomes in CRC patients. Estrogen is known to modulate the TME, enhancing anti-tumor immunity and inhibiting tumor growth. However, a gap exists as current research has not yet leveraged single-cell analyses to explore the heterogeneity of the TME and its distinct impacts on CRC development between sexes. Furthermore, the existing body of research neglects the intricate cellular crosstalk among diverse elements within the TME. Our study addresses this gap by employing single-cell RNA transcriptome sequencing to comprehensively analyze sex disparities in the CRC TME. At single-cell resolution, we examine the differences in immune cells, stromal cells, and cancer cells regarding composition and function. This analysis elucidates the components of the TME that contribute to sex-specific disparities in CRC survival rates, potentially unveiling targets for precision therapy in the prevention and treatment of colorectal cancer.

## Materials and Methods

### Human subjects

After obtaining approval from the Ethics Committee of the Sixth Affiliated Hospital of Sun Yat-sen University (No.2024ZSLYEC-240), this study enrolled 32 CRC patients diagnosed at the Sixth Affiliated Hospital of Sun Yat-sen University were enrolled in this study, including 19 males and 13 females. No statistically significant differences in age were observed between the sexes (p = 0.7484), tumor stage (p = 0.8758), or histological type (p = 0.5427). Detailed clinical and pathological information are presented in Table S1.

### Single-cell suspensions, library construction, and sequencing

Fresh specimens of tumor tissues were carefully cleaned with Dulbecco's Phosphate-Buffered Saline first and then cut into 1-2 mm^3^ cubes on ice, while kept on ice. Enzymatic digestion was conducted using the MACS Human Tumor Dissociation Kit (Miltenyi Biotec) on these tissue fragments. Single-cell 3'-libraries were prepared using the DNA Nanoball (DNB) elab C4 scRNA Preparation Kit in following the manufacturer's protocol. After library construction, sequencing was performed on the DNBelab C4 sequencing platform, and the raw reads were processed with stringent filtration and demultiplexing using the PISA software for accurate data analysis (https://github.com/shiquan/PISA).

### Single-cell RNA-seq data processing

The refined sequencing reads aligned to the human genome using the STAR (v.2.7.4a), and sorted with Sambamba (v.0.7.0). The resulting cell-gene count matrix was then imported into Seurat R package (v.4.2.2) to create a Seurat object, ready for subsequent analysis 16.

To ensure the quality of our downstream analysis, we implemented stringent filtering criteria to select only high-quality cells. We excluded cells based on the following criteria: 1. Cells with more than 20% mitochondrial transcripts, indicating potential cell stress or damage. 2.Cells expressing fewer than 300 genes or more than 6,000 genes, which could represent low-quality or highly variable cells. 3.Doublets and contaminant cells identified by disordered clustering in the UMAP embedding space or chaotic marker expression. 4.Cells with high read depths exceeding 30,000 UMI counts, which may indicate technical artifacts or over-amplification. After filtering, we normalized the discrete gene expression counts across individual cells within each sample using the "LogNormalize" function from Seurat. This normalization step helps to reduce the impact of gene expression variability due to differences in sequencing depth and allows for more accurate comparisons between cells. Next, we employed the "FindVariableFeatures" function from Seurat (v.4.2.2) to identify the top 3,000 genes with high variability, termed Highly Variable Genes. These HVGs are crucial for capturing the biological variability within the dataset and are essential for subsequent dimensionality reduction and clustering analyses. For UMAP projection and clustering analysis, we utilized the top 30 principal components and set a resolution of 0.4.

### Cell type annotation

We identified various cell types, including B cells, plasma cells, epithelial cells, endothelial cells, myeloid cells, fibroblasts, pericytes, mast cells, T cells, subsets of T cells, and subsets of myeloid cells, using classic cell-type markers. Subsets of tumor cells, B cells, myeloid cells, and fibroblasts were further classified based on differentially expressed genes using Seurat's "FindAllMarkers" function.

### Gene signature score

We employ the R package AUCell 17 (v1.20.2) to assess cellular functions and signaling pathways on the normalized matrix of Seurat objects. The gene lists for this analysis are provided in Table S2. We utilized the myeloid cell function gene list from Sun et al 18 in Figure 5D and the T-cell cytotoxic and proliferative score gene list from Huang et, al 19 in Figure 3C, G.

### Pathway Enrichment analysis

Using the clusterProfiler 20 (v4.6.2) and GSEABase 21 (v1.60.0) R packages, we performed a comparative pathway enrichment analysis on the Seurat object to identify sex-specific differences in enriched pathways. Significance was ascertained with a stringent p cutoff of less than 0.05.

### Cell-cell interaction analysis

Using the CellChat 22 (v1.6.1) R package, we conducted an intercellular communication analysis to explore the interactions between distinct cell types. Following the official workflow, we transformed Seurat objects into CellChat objects. We then calculated ligand-receptor pairs and their communication probabilities based on the CellChatDB human database. Ligands and receptors expressed in fewer than ten cells within a cell type were excluded from the analysis.

### Transcription factor and gene regulatory network analysis

We utilized the SCENIC 17 (v1.1.2.2) R package to analyze the regulatory networks involving transcription factors (TFs) and their target genes. By quantifying gene regulatory networks (GRNs) at the single-cell level, we mapped the regulatory interactions between TFs and their targets. Each regulatory module was assigned AUC scores to assess their activity. The analysis aimed to identify TFs with significant regulatory impact across different cell types and sexes by evaluating the regulatory intensity of TFs and their target genes. This approach helps to uncover the key regulatory elements that may contribute to sex-specific differences in cellular behavior within the tumor microenvironment.

### Developmental trajectory analysis

We employed Monocle2 23 algorithm for cell trajectory analysis, utilizing the "DDRTree" method to perform dimensionality reduction and order cells based on differentially expressed genes specific to B cell subsets. Additionally, we applied the Cytotrace 24 algorithm to predict cell ordering, adhering to its official workflow for accurate trajectory inference.

### Ro/e tissue preference analysis

We used a well-established approach from prior literature 25 to assess sex-based tissue preference by calculating the observed-to-expected ratios (Ro/e) for various cell types. A Ro/e ratio > 1 indicated enrichment of that cell type in a particular sex. "+++" stands for Ro/e score > 1, "++" stands for Ro/e score ≤ 1 but > 0.8. "+" stands for Ro/e score ≤ 0.8 & ≥ 0.2. "+/-" stands for Ro/e score ≤ 0.2 & > 0. "-" stands for Ro/e score = 0. This quantification helped us identify cell types with significant sex-based differences in tissue distribution.

### Survival analysis

To perform single-gene survival curve analysis, bulk RNA-seq data, including clinical information and gene expression matrices, were obtained from the Cancer Genome Atlas Program (https://www.cancer.gov/ccg/research/genome-sequencing/tcga). The optimal cutoff value for gene expression was determined using the "surv_cutpoint" function from the survminer (v0.4.9) package. Survival curves were then generated with the "survfit" function from the survival (v3.4-0) package and visualized using the ggsurvplot function.

### Multi-color immunohistochemistry

We conducted multi-color immunohistochemistry on paraffin-embedded CRC sections collected from the Sixth Affiliated Hospital of Sun Yat-sen University. The cohort consisted of 5 male and 5 female CRC cases, none of which had undergone prior neoadjuvant therapy.

The paraffin sections were first heated in an oven at 65℃ for one hour, then deparaffinized in xylene, followed by rehydration through a graded series of solutions, including anhydrous ethanol, 95% ethanol, 75% ethanol, and distilled water. Staining was performed according to the manufacturer's protocol using the PANO 5-plex IHC Kit (Cat#10002100100, Panovue). Antigen retrieval was carried out by heating the sections in citrate solution (pH 9.5, ZSGB-BIO) in a pressure cooker for 18 minutes. Following this, the sections were incubated with blocking buffer at 37℃ for 10 minutes. Rabbit anti-human EOMES (Abcam, clone EPR21950-241, 1:50) was applied and incubated overnight at 4℃, followed by the addition of a secondary horseradish peroxidase-conjugated antibody (Panovue) and incubation at 37℃ for 10 minutes. Signal amplification was performed using the TSA working solution, diluted 1:100 in amplification diluent (Panovue), with a 10-minute incubation at 37℃. Subsequently, rabbit anti-human GZMB (CST, clone D6E9W, 1:200) and mouse anti-human CD8 (CST, clone C8/144B, 1:200) were incubated at 37℃ for 1 hour, with the subsequent steps following the same protocol. Finally, nuclei were stained with DAPI. Image acquisition was performed using the TissueFAXS cytometry platform, and images were analyzed using StrataQuest software.

### Cell culture and treatment

Human LS174T (#STCC10816, Servicebio) and human RKO CRC cell lines (#STCC00054P-1, Servicebio) were cultured in Roswell Park Memorial Institute-1640 medium (Corning) supplemented with 10% fetal bovine serum (Procell), and 1% penicillin and streptomycin (ThermoFisher) at 37℃ in a humidified 5% CO_2_ chamber. 1Mβ-estradiol (E2, #50-28-2, Merck) was dissolved in 1 mL of anhydrous ethanol by gentle rotation. Subsequently, 49 mL of sterile culture medium was added to the solution, and the mixture was serially diluted to final concentrations of 10⁻⁶ M, 10⁻⁷ M, and 10⁻⁸ M. After incubation with these estrogen concentrations or vehicle (2% ethanol) for 48 hours, cells were subjected to Western blot and TUNEL staining to assess the level of apoptosis.

### Western blot

LS174T cells and RKO cell lines were each set up with control groups, E2-treated groups (10⁻⁶ M, 10⁻⁷ M, and 10⁻⁸ M). After incubation with E2 for 48 hours, proteins were extracted from the cells. The protein concentration of the samples was determined using a BCA assay kit (#G2026, Servicebio). Total proteins were then separated by 10% SDS-PAGE (Vazyme) and transferred onto PVDF membranes (#ISEQ00010, Millipore). The membranes were blocked with a rapid blocking solution (#G2052, Servicebio) and subsequently incubated overnight at 4℃ with primary antibodies against BAX (#D2E11, 1:1000, Cell Signaling Technology), BCL-2 (#R22494, 1:1000, ZEN BIO), CASP9 (#R22844, 1:1000, ZEN BIO), Cleaved-CASP9 (Cle-CASP9, #R381336, 1:1000, ZEN BIO) and GAPDH (#ZB15004-HRP-100, 1:3000, Servicebio). The membranes were then incubated with HRP-conjugated goat anti-rabbit IgG (#GB23303, 1:10000, Servicebio). Finally, the proteins were visualized using ECL reagent (#G2020, Servicebio). Semi-quantitative analysis was performed using ImageJ software.

### TUNEL staining

Cell apoptosis was assessed by TUNEL staining using the TMR (red) TUNEL Cell Apoptosis Detection Kit (#G1502, Servicebio). RKO and LS174T cell lines were plated at a density of 1×10⁵ cells per 35mm dish. Cells were fixed with 4% paraformaldehyde solution (dissolved in PBS) and permeabilized with Proteinase K for 10 minutes. Positive controls were prepared by treating samples with DNase I (#G3342, Servicebio). The TdT incubation buffer was prepared according to the manufacturer's instructions. After incubation in the dark for 1 hour, nuclei were stained with an anti-fade mounting medium containing DAPI (#G1407, Servicebio), and images were captured using a Pannoramic 250 FLASH III Digital Scanner.

### Statistics and Reproducibility

We utilized unpaired two-tailed Wilcoxon rank-sum tests to assess differences in cell distribution between sexes. One-way ANOVA using the two-stage linear step-up procedure by Benjamini, Krieger, and Yekutieli was employed to determine inter-group differences during multiple comparisons. Pearson correlation coefficients were employed for correlation analysis to examine the relationships between genes and gene sets, as well as between different gene sets. p < 0.05 is considered statistically significant. All statistical analyses and data presentations were performed by the R program (v 4.2.2).

## Results

### Sex-specific disparities in the CRC Tumor Microenvironment

**Figure 1:**
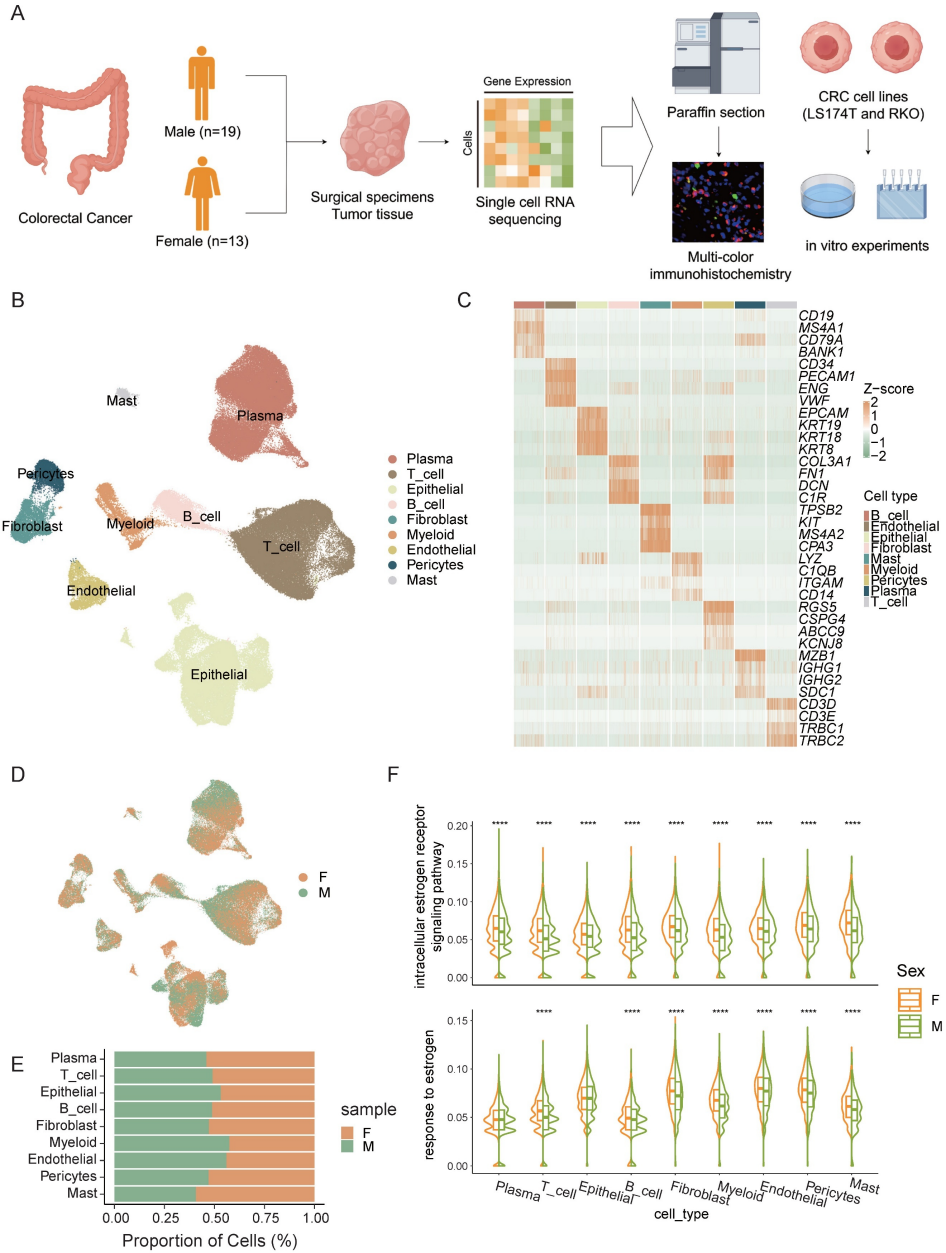
**Sex-biased disparities in the CRC Tumor Microenvironment. A)** Schematic workflow of experimental design and data analysis. **B)** UMAP plot of the major cell types. **C)** The normalized expression of the marker genes of cell types. **D)** UMAP plot of female CRC (F) and male CRC (M). **E)** Bar plot shows the proportion of different sexes in the cell types. **F)** Gene set scores of estrogen response pathways from GO: BP database among different sexes in cell types. Significance levels are expressed as *p<0.05, **p<0.01, ***p<0.001, and ****p<0.0001.

To explore sex-specific disparities in the tumor microenvironment of CRC, we performed single-cell sequencing on surgical specimens or endoscopic biopsies from 32 CRC patients, including 19 males and 13 females (Figure 1A). From the 32 samples, we collected a total of 256,971 cells, and after stringent quality control measures, 167,437 cells were retained for subsequent analysis.

We identified 9 distinct cell types (Figure 1B), including T cells, B cells, plasma cells, epithelial cells, endothelial cells, myeloid cells, fibroblasts, pericytes, and mast cells based on their expression of marker genes (Figure 1C). Significant heterogeneity in cell type composition was observed between the sexes (Figure 1D, E). Differences in estrogen response pathways across various cell types were validated, revealing marked disparities in estrogen responses in most cell populations (Figure 1F). These findings suggest that estrogen plays a crucial role in shaping the tumor microenvironment and may be a major factor driving sex -specific disparities in the immune landscape of colorectal cancer.

### Estrogen promotes the apoptosis of cancer cells

**Figure 2:**
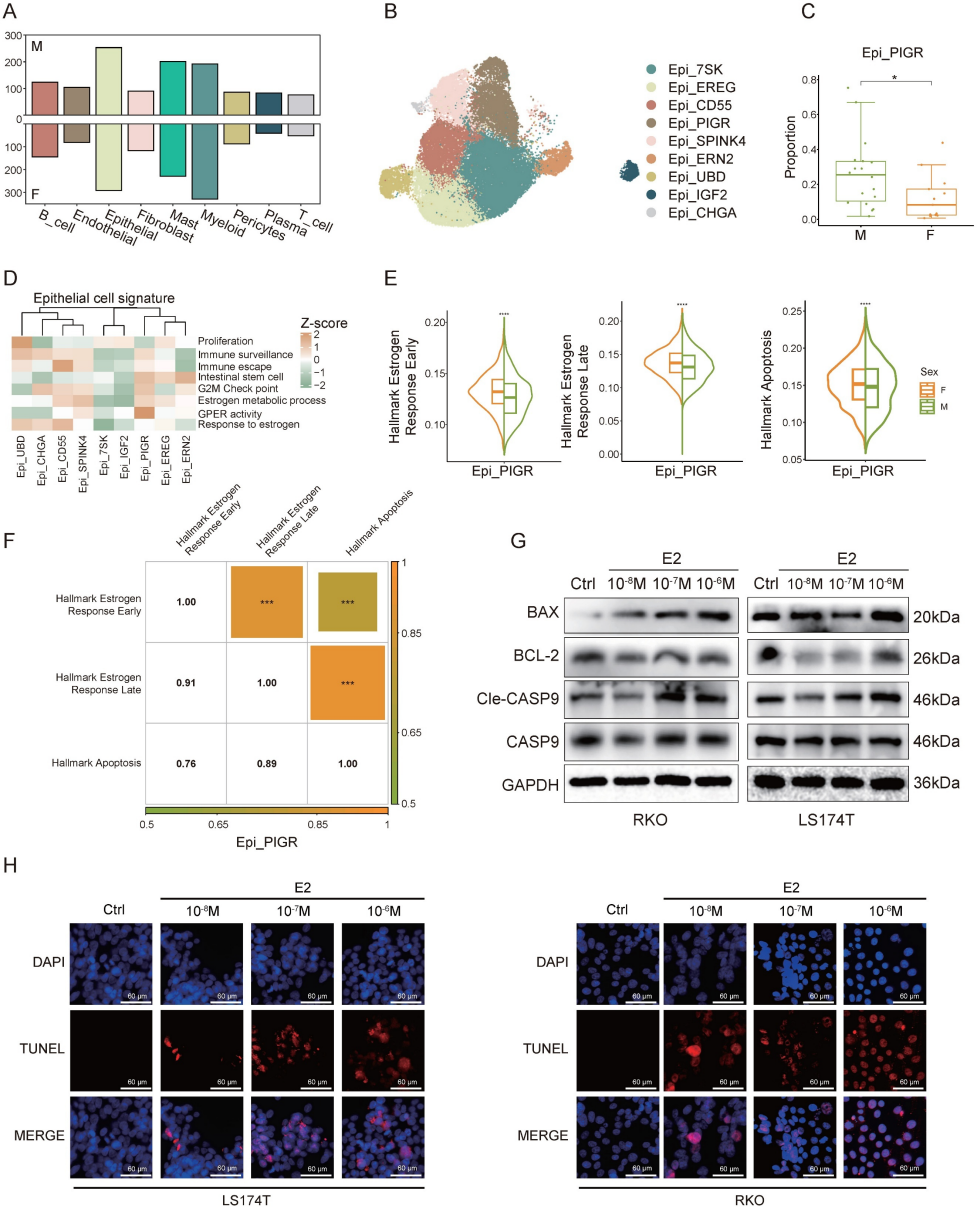
**Estrogen promotes apoptosis in cancer cells. A)** Bar plot shows the number of different expressed genes of female CRC (F) and male CRC (M). **B)** UMAP plot of subsets of epithelial cells. **C)** Proportion comparison of Epi_PIGR between female CRC (F) and male CRC (M). Two-sided Wilcoxon test. Significance levels are expressed as *p<0.05, **p<0.01, and ***p<0.001. **D)** Gene set scores among epithelial subsets. Gene set of Proliferation, Immune_surveillance, Immune_escape, Intestinal_stem_cell, and G2M_Check_point is listed in table S2. Estrogen and GPCR pathways from GO: BP database is also used. **E)** Violin plots show gene set scores of Hallmark pathways in Epi_PIGR between female CRC (F) and male CRC (M). Significance levels are expressed as *p<0.05, **p<0.01, ***p<0.001, and ****p<0.0001. **F)** Heatmap shows the correlation coefficients of gene set scores of Hallmark pathways in Epi_PIGR. Significance levels are expressed as *p<0.05, **p<0.01 and ***p<0.001. Pearson correlation coefficient. **G)** Western blot analysis of protein expression levels of BAX, BCL-2, CASP9, Cle-CASP9, and GAPDH in RKO and LS174T cell lines following treatment with varying concentrations of E2 (10^-6^ M, 10^-7^ M, 10^-8^ M) and control (Ctrl) conditions. **H)** TUNEL staining in RKO and LS174T cell lines following treatment with varying concentrations of E2 (10^-6^ M, 10^-7^ M, 10^-8^ M) and control (Ctrl) conditions. Scale bar: 60 μm.

Differential gene expression analysis across various cell types between sexes revealed the most pronounced sex-specific differences in epithelial cells (Figure 2A). To investigate these differences further, we performed a clustering analysis of epithelial cells (Figure 2B). We found that Epi_PIGR showed a significant sex-based proportion difference (Figure 2C). Gene set scoring revealed that Epi_PIGR exhibits intestinal stem cell characteristics and strong G protein-coupled estrogen receptor (GPER) activity (Figure 2D). Prior studies have indicated that GPER, upon estrogen binding, can induce CRC cell apoptosis 26.

We compared signature scores of estrogen response and apoptosis pathways in Epi_PIGR between sexes and found elevated expression in female CRC patients (Figure 2E). Correlation analysis further revealed significant associations among these pathways, suggesting estrogen might induce cancer cell apoptosis in CRC (Figure 2F), which could account for the sex difference in Epi_PIGR abundance. A statistically significant correlation among these pathways was also observed across the entire epithelial cell population (Figure S1A). To investigate the estrogen-mediated apoptotic effects on tumor cells, we performed a series of *in vitro* experiments using two CRC cell lines (LS174T and RKO). Estrogen treatment induced a significant reduction in the anti-apoptotic protein BCL-2, while simultaneously increasing the levels of the pro-apoptotic protein BAX, compared to untreated controls. Similarly, CASP9 was activated by estrogen and converted to Cle-CASP9 (Figure 2G, Figure S1B). We further conducted TUNEL assays to evaluate DNA fragmentation in CRC, revealing that estrogen increased the proportion of apoptotic cells (Figure 2H). These findings collectively demonstrate estrogen-induced apoptosis in CRC.

In summary, our findings reveal sex-specific differences in estrogen response, and apoptosis pathways, indicating a potential role for estrogen in promoting cancer cell apoptosis, which may impact CRC prognosis.

### The sex-specific differences in the EOMES transcription module affect the anti-tumor functions of CD8+ T cells

**Figure 3:**
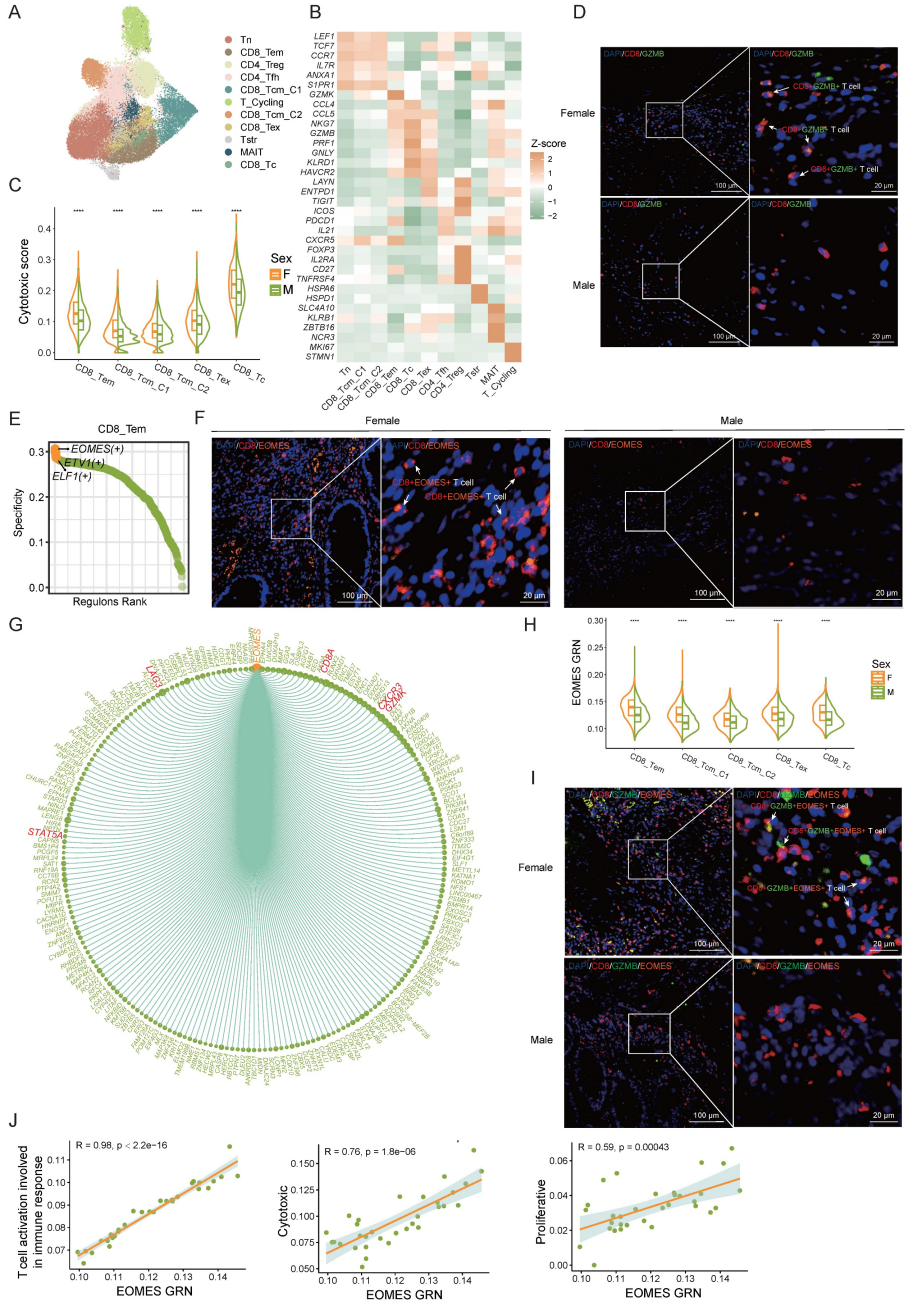
**The sex-specific differences in the EOMES transcription module affect the anti-tumor functions of CD8+ T cells. A)** UMAP plot of subsets of T cells. **B)** The normalized expression of the marker genes of T-cell subsets. **C)** Cytotoxic gene set scores among CD8+ T sub types between female CRC (F) and male CRC (M). Significance levels are expressed as *p<0.05, **p<0.01, ***p<0.001, and ****p<0.0001. **D)** Multicolor IHC staining of CD8+ GZMB+ T cells in one representative female or male CRC tumor. Scale bar: 100 μm and 20 μm. **E)** Subset-specific transcription factor of CD8_Tem. **F)** Multicolor IHC staining of CD8+ EOMES+ T cells in one representative female or male CRC tumor. Scale bar: 100 μm and 20 μm. **G)** Chord plot shows EOMES (+) gene regulatory network (GRN). **H)** Violin plots show gene set scores of EOMES (+) GRN in CD8+ T cells between female CRC (F) and male CRC (M). Significance levels are expressed as *p<0.05, **p<0.01, ***p<0.001, and ****p<0.0001. **I)** Multicolor IHC staining of CD8+ GZMB+ EOMES+ T cells in one representative female or male CRC tumor. Scale bar: 100 μm and 20 μm. **J)** Correlation plots of gene set scores of EOMES (+) GRN and T cell function in CD8+ T cells.

T cells play a pivotal role in governing tumor growth and development as a key component of the defense mechanism. We performed clustering analysis of T cells and further annotated them according to marker genes associated with various T cell subsets (Figure 3A, B). Our analysis identified several T cell populations, including naive T cells, CD8+ T cells, CD4+ T cells, MAIT cells, cycling T cells, and stress response T cells (Tstr). CD8+ T cells were further classified into CD8_Tem (CD8+ effector memory T cells), CD8_Tcm_C1 (CD8+ central memory T cells), CD8_Tcm_C2, CD8_Tc (CD8+ cytotoxic T cells), and CD8_Tex (CD8+ exhausted T cells) subsets, while CD4+ T cells included CD4_Tfh (CD4+ follicular helper T cells) and CD4_Treg (CD4+ regulatory T cells) subsets. Consistent with existing literature 13, we observed that CD4+ and CD8+ T cells were more abundant in females, whereas naive T cells and Tstr cells were predominantly present in males. (Figure S2A).

To assess sex differences in CD8+ T cell anti-tumor function, we evaluated cytotoxic gene set scores in CD8+ T cell subsets by sex. The cytotoxicity score of CD8+ T cells in female CRC patients was significantly higher than in males (Figure 3C) , aligning with previous research 27. In our multi-color immunohistochemistry cohort, we also observed a higher proportion of CD8+ GZMB+ T cells in females (Figure 3D, Figure S2B).

To delve into the factors behind sex differences in CD8+ T cell cytotoxicity, we employed the SCENIC algorithm to analyze gene regulatory network (GRN) of transcription factors in CD8+ T cells. EOMES emerged as the most specific transcription factor in CD8_Tem cells (Figure 3E). Prior research underscores EOMES's role in CD8+ T cell differentiation and anti-tumor immunity 28-31. Multi-color immunohistochemical analysis revealed significantly higher EOMES expression in female CD8+ T cells compared to males (Figure 3F, Figure S2C). The EOMES (+) GRN includes 218 downstream target genes such as CD8A, GZMK, STAT5A, CXCR3, and LAG3 (Figure 3G). Sex comparison of the EOMES (+) GRN activity showed higher expression in female CD8+ T cells (Figure 3H). Multi-color immunohistochemistry also indicated higher EOMES levels in female CD8+ GZMB+ T cells (Figure 3I, Figure S2D). Correlation analysis revealed significant associations between EOMES (+) GRN expression and CD8+ T cell activation pathways, proliferation scores, and cytotoxicity scores (Figure 3J), suggesting sex differences in the EOMES (+) GRN might affect the activity and tumor-killing function of CD8+ T cells, thereby influencing anti-tumor immunity.

In conclusion, our results support stronger CD8+ T cell activity in female CRC patients and identify the EOMES GRN as a key factor in sex-based T cell cytotoxicity disparities.

### Sex-specific functional differences in B cell antigen presentation

**Figure 4:**
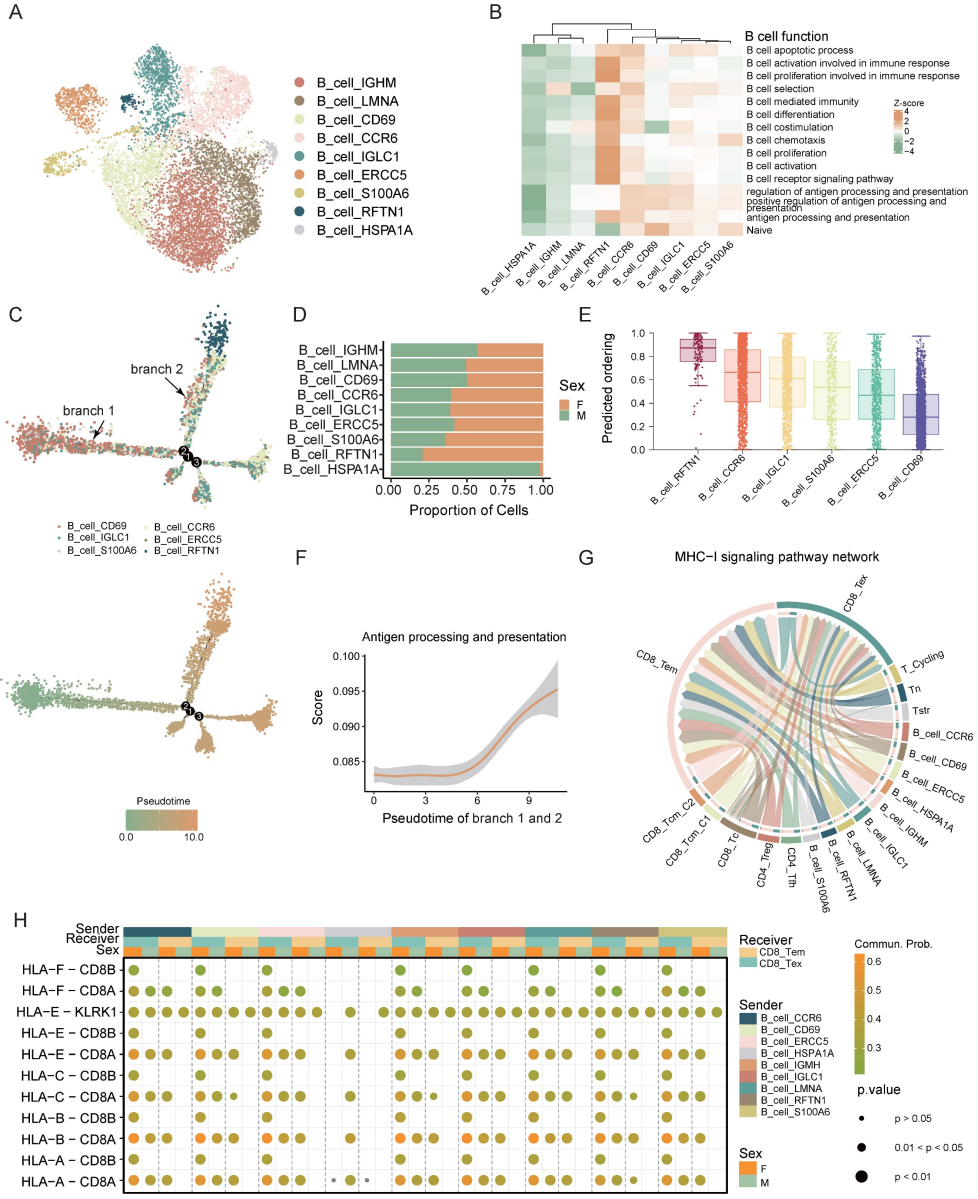
** Sex-biased functional differences in B cell antigen presentation. A)** UMAP plot of subsets of B cells. **B)** Gene set scores of B cell function from GO: BP database among B cell subsets. **C)** Developmental trajectory of B cell subsets grouped by cell type (up) and pseudotime (bottom). **D)** Bar plot shows the proportion of different sexes in the B cell subsets. **E)** Boxplots show the predicted ordering of B cell subsets. **F)** Gene set score of “Antigen processing and presentation” from GO: BP database of B cell developmental trajectory. **G)** Chord plot shows the cell-cell interaction of MHC-I signaling pathway network between B cells and T cells. **H)** Bubble plot shows the cell-cell interaction of MHC-I signaling pathway network between B cells and T cells of different sexes.

B cells are essential components of the immune system, primarily responsible for antigen presentation. We performed a clustering analysis of B cells and identified 9 distinct B cell subsets: B_cell_IGHM, B_cell_LMNA, B_cell_CCR6, B_cell_CD69, B_cell_IGLC1, B_cell_ERCC5, B_cell_S100A6, B_cell_HSPA1A, and B_cell_RFTN1. These subsets were designated based on their differential gene expression patterns (Figure 4A).

We performed gene set scoring for each subset to elucidate their functional states, identifying B_cell_RFTN1 as the most activated subset among B cells (Figure 4B).

We conducted trajectory analysis focusing on the relatively activated B-cell subsets, specifically B_cell_CD69, B_cell_CCR6, B_cell_IGLC1, B_cell_ERCC5, B_cell_S100A6, and B_cell_RFTN1 (Figure 4C). This analysis revealed a developmental trajectory initiating from B_cell_CD69 and ending at B_cell_RFTN1. Proportion comparison showed that mature B cells (B_cell_RFTN1) are predominantly found in females, whereas the relatively immature B_cell_CD69 is more prevalent in males (Figure 4D). Utilizing both Cytotrace predicted ordering and gene set scoring, we ascertained that B_cell_CD69 denotes the most immature state within the B-cell lineage, while B_cell_RFTN1 signifies the highest maturation stage (Figure 4B, E). Additionally, our analysis revealed a progressive enhancement in the antigen-presenting capacity of B cells during their differentiation trajectory. (Figure 4F). Pathways associated with antigen presentation is found to be particularly enriched in B_cell_RFTN1 and it exhibited elevated expression levels of major histocompatibility complex (MHC) related genes (Figure S3A, B).

Using the CellChat algorithm, we analyzed MHC-I signaling pathway interactions between B and T cells, revealing extensive antigen presentation from B cells to CD8+ T cells (Figure 4G). Furthermore, the female tumor microenvironment also demonstrated enhanced MHC-I interactions between B cells and CD8+ T cells (Figure 4H). These findings suggest that B cells exhibiting advanced differentiation and heightened antigen-presenting capabilities are more prevalent in female CRC patients. This sex disparity in antigen presentation efficacy between B and T cells within the tumor microenvironment could contribute to the observed sex-based variations in CRC prognosis.

In conclusion, our analysis revealed sex-specific variations in B cell differentiation and antigen presentation, potentially contributing to the improved prognosis observed in female CRC patients.

### Macrophages interact with CD8+ T cells via the TNF-TNFRSF1B ligand-receptor pair, showing higher intensity in female CRC

**Figure 5:**
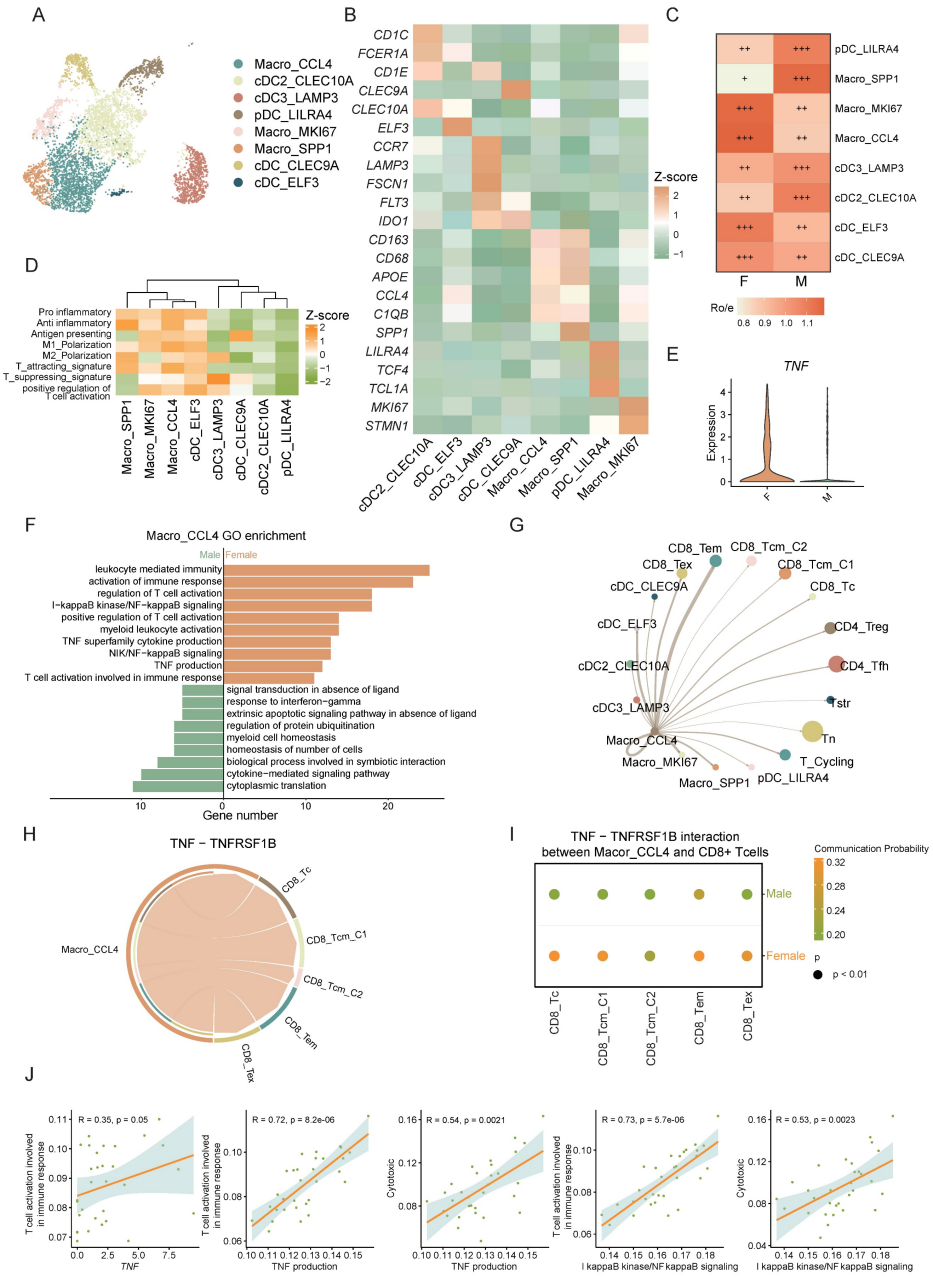
** Macrophages interact with CD8 T cells via the TNF-TNFRSF1B ligand-receptor pair, showing higher intensity in female CRC. A)** UMAP plot of subsets of myeloid cells. **B)** The expression of the marker genes of myeloid subsets. **C)** Tissue prevalence of myeloid subsets estimated by Ro/e score, in which Ro/e denotes the ratio of observed to expected cell number. "+++" stands for Ro/e score > 1, "++" stands for Ro/e score ≤ 1 but > 0.8. "+" stands for Ro/e score ≤ 0.8 & ≥ 0.2. "+/-" stands for Ro/e score ≤ 0.2 & > 0. "-" stands for Ro/e score = 0. **D)** Gene set scores of myeloid cell function among myeloid subsets. **E)** Violin plot shows the TNF expression of different sexes in Macro_CCL4.** F)** GO pathway enrichment of different sexes in Macro_CCL4. **G)** Chord plot shows the cell-cell interaction from Macro_CCL4 to other myeloid subsets and T cells. **H)** Chord plot shows the cell-cell interaction of TNF-TNFRSF1B between Macro_CCL4 and CD8+ T cells.** I)** Bubble plot shows the cell-cell interaction of TNF-TNFRSF1B between Macro_CCL4 and CD8+ T cells of different sexes. **J)** Correlation plots of TNF expression or TNF related pathway scores in Macro_CCL4 and gene set scores CD8+ T cells.

Macrophages are crucial for immune regulation within the tumor microenvironment. We distinguished myeloid cells into macrophages and dendritic cells (DCs) based on marker gene expression. Macrophage subsets were Macro_CCL4, Macro_SPP1, and Macro_MKI67, while DC subsets included cDC_CLEC10A, cDC_LAMP3, pDC_LILRA4, cDC_CLEC9A, and cDC_ELF3. (Figure 5A, B).

The Macro_CCL4 subset exhibited a higher tissue prevalence in females (Figure 5C) and was significant enriched in immune-related pathways highlighting its pivotal role in T cell recruitment and activation (Figure 5D, Figure S4A). TNF, a critical cytokine in the tumor immune microenvironment, plays a multifaceted role in tumor initiation, progression, and anti-tumor immunity 32. NF-κB is crucial for inducing many cytokines and immune-regulatory proteins in TNF-mediated diverse biological responses 33. IκB kinase (IKK), a regulator of NF-κB, triggers its release by phosphorylating the inhibitor of NF-κB (IκB) 34. Studies suggest that low levels of estrogen enhance cytokine expression like TNF in monocytes and macrophages 10. In our study, Macro_CCL4 was found to be enriched in TNF related pathways including TNF production and I-kappaB kinase/NF-kappaB signaling (Figure S4A). Further analysis between sexes revealed that the TNF related pathways are predominantly enriched in the female-derived Macro_CCL4 subset compared to males, with significant sex differences in TNF expression (Figure 5E, F). GSEA further confirmed the significant activation of TNF signaling pathway in female-derived Macro_TNF cell aligning with our prior findings (Figure S4B).

To elucidate Macro_CCL4's role in immune modulation, we employed the CellChat algorithm to deduce the communication between Macro_CCL4 and T cells. Our analysis indicated substantial crosstalk between Macro_CCL4 and T cells, suggesting a complex network of interactions crucial for immune regulation (Figure 5G). TNFRSF1B (also known as TNFR2), part of the TNF receptor superfamily TRAF (TNF receptor-associated factor) interaction subgroup, co-stimulates CD8+ T cells and sensitizes tumor cells to TNFR1-mediated cytotoxicity 35, and is instrumental for CD8+ T cell activation and cytotoxicity during early immune responses. By lowering TCR signaling thresholds, it significantly impacts CD8+ effector T cell function 36-38. Our findings show extensive Macro_CCL4 - CD8+ T cell interactions via the TNF-TNFRSF1B axis, which is enhanced in female CRC patients (Figure 5H, I).

Further investigation into Macro_CCL4 and CD8+ T cell interaction revealed significant correlations between TNF expression in Macro_CCL4 and CD8+ T cells activation pathways. The TNF related pathways in Macro_CCL4 also correlated strongly with CD8+ T cell activation and cytotoxicity scores (Figure 5J, Figure S4C). It intimates a potential role for TNF secreted by Macro_CCL4 in modulating T cell activation and enhancing the cytotoxic capacity against tumor cells. This implicates that Macro_CCL4-derived TNF might be instrumental in orchestrating the immune response within the tumor microenvironment.

Altogether, these findings indicate that sex-specific interactions of Macro_CCL4 with CD8+ T cells via TNF-TNFRSF1B may contribute to sex disparities in the prognosis of CRC.

### The male-dominant CAF_MMP11, characterized as the mCAF phenotype, promoting CRC progression through multiple interactions

**Figure 6:**
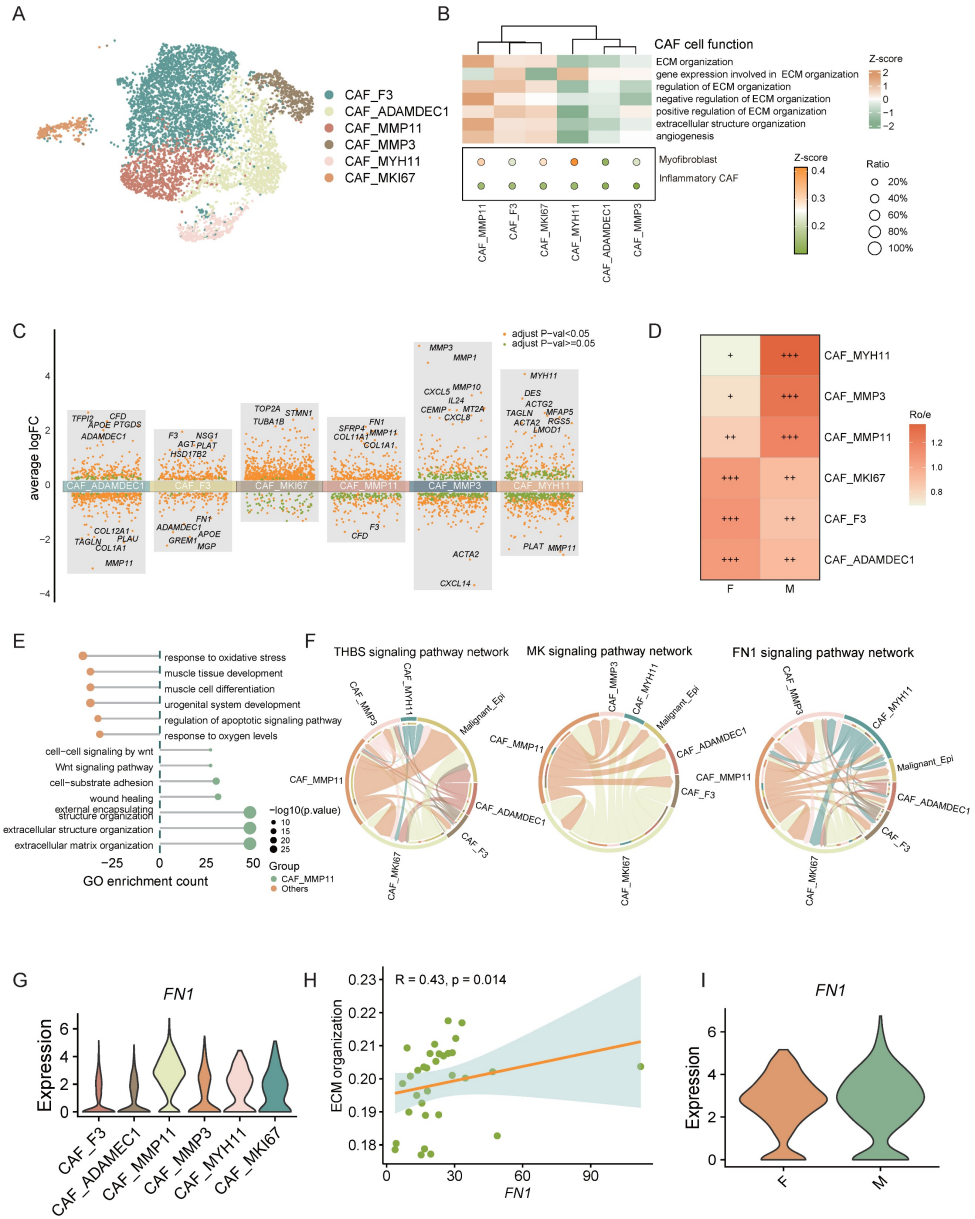
** The male-dominant CAF_MMP11, characterized as the mCAF phenotype, promotes CRC progression through multiple interactions. A)** UMAP plot of subsets of CAFs. **B)** Gene set scores of GO: BP pathways (heatmap) and myofibroblastic/inflammatory CAF (bubble plot) among CAF subsets.** C)** Volcano plot shows the different expressed genes of every CAF subset comparing to other subsets. **D)** Tissue prevalence of CAF subsets estimated by Ro/e score, in which Ro/e denotes the ratio of observed to expected cell number. "+++" stands for Ro/e score > 1, "++" stands for Ro/e score ≤ 1 but > 0.8. "+" stands for Ro/e score ≤ 0.8 & ≥ 0.2. "+/-" stands for Ro/e score ≤ 0.2 & > 0. "-" stands for Ro/e score = 0. **E)** GO pathway enrichment of CAF_MMP11 comparing to other CAF subsets. **F)** Chord plot shows the cell-cell interaction of THBS, MK, and FN1 signaling pathway network between CAF subsets and Tumor cells. **G)** Violin plot shows gene expression of FN1 in CAF subsets. **H)** Correlation plot of FN1 expression and gene set score of ECM organization pathway in CAF_MMP11. I) Violin plot shows gene expression of FN1 in CAF_MMP11 between female CRC (F) and male CRC (M).

Cancer-associated fibroblasts (CAFs) are essential stromal components of stromal cells, playing pivotal roles in promoting tumor progression. Through single-cell clustering and annotation of fibroblasts, we identified 6 distinct cell types: CAF_F3, CAF_MMP11, CAF_MYH11, CAF_MKI67, CAF_MMP3, and CAF_ADAMDEC1 (Figure 6A). Current understanding divides CAFs into two main groups with unique TME roles: myofibroblastic CAFs (mCAFs), involved in ECM remodeling and metastasis, and inflammatory CAFs (iCAFs), which modulate immunity and recruit immune cells, thus influencing the tumor's immune context, highlighting their role in shaping the immune landscape within the tumor 39-43. Gene set scoring for ECM remodeling and different expressed genes identified CAF_MKI67, CAF_MMP11, and CAF_MYH11 as mCAFs (Figure 6B, C). Notably, CAF_MMP11 showed the strongest ECM remodeling function, with elevated expression of MMP11 and FN1, key players in ECM dynamics (Figure 6B, C). MMP11, from the metalloproteinase family, aids in ECM degradation and tissue remodeling 44 , while FN1 is vital for cell adhesion and migration, essential for cancer progression 45. Both of these factors are crucial in ECM remodeling and tumor metastasis. CAF_MMP11 was more prevalent in male CRC, suggesting a link to sex-specific tumor traits (Figure 6D). GO pathway enrichment analysis confirmed CAF_MMP11's role in ECM-related pathways, highlighting its contribution to tumor progression and metastasis (Figure 6E).

Moreover, cell interaction analysis between CAFs and malignant epithelial cells revealed that CAF_MMP11, an mCAF subset, interacts with these cells via signaling pathways such as THBS, MK, and FN1 (Figure 6F). Studies indicates that CAF-derived THBS2 promotes tumor growth and adhesion 46. CAF-derived MK may also enhance tumor resistance through various mechanisms 47, 48. FN1 knockdown in CAFs diminishes cancer cell migration, and FN1 promotes tumor metastasis 49, 50. These findings suggest that mCAFs in the CRC tumor microenvironment can promote tumor metastasis and progression through various mechanisms.

We further analyzed the ligands engaged in these interactions and determined that the majority of FN1 was derived from CAF_MMP11. Moreover, correlation analysis showed a strong positive link between FN1 expression and the ECM organization ability of CAF_MMP11. Further investigation demonstrated that FN1 expression was markedly upregulated in male CAF_MMP11. These findings collectively imply that FN1 might be a crucial driver for CAF_MMP11 in ECM remodeling, thereby contributing to tumor metastasis in male CRC.

In summary, we identified pro-tumorigenic CAF_MMP11 in the CRC tumor microenvironment, which are more prevalent in males and likely promote tumor progression by modulating cell-cell interactions and ECM remodeling, indicating a sex-disparate distribution.

## Discussion

CRC, a prevalent digestive tract malignancy, ranked third globally among malignancies in 2020 based on incidence and second in terms of mortality 1. Notably, men show higher incidence and mortality rates than women in every age group from 50 to 75 3. However, the biological mechanisms behind sex disparities in CRC prognosis remain inadequately explored and necessitate further investigation.

Multiple factors contributing to sex differences may influence CRC prognosis. For instance, female CRC tends to occur more frequently in the right-sided colon, while males have a higher red meat consumption 4, 7. The tumor microenvironment, consisting of cancer cells, immune cells, and stromal components, engages in intricate crosstalk among different cell types, impacting the tumor-immune balance and potentially influencing CRC development and progression.

Previous research has primarily focused on sex differences in T cells in CRC, revealing that females have a higher prevalence of CD4+ and CD8+ T cells, with CD8+ T cells showing more stemness traits 12, 13. Estrogens also modify the tumor microenvironment by enhancing tumor cell apoptosis and reducing PD-L1 expression, impacting CRC prognosis. However, the role of sex disparities in other tumor microenvironment cell types and the effects of estrogens require further investigation. Our study leverages single-cell transcriptomic analysis to unravel profound sex-specific disparities in the TME of CRC, offering mechanistic insights into the differential immune and stromal responses that underlie prognostic variations between males and females.

We found a significant positive correlation between the estrogen pathway and apoptosis levels through single-cell data analysis, consistent with previous research 26. The validation of estrogen-induced apoptosis further underscores hormonal regulation as a cornerstone of sex differences, with estrogen suppressing BCL-2 and activating caspase cascades. The enhanced cytotoxic activity of CD8+ T cells in female CRC aligns with prior reports of sex-biased T cell functionality 12, 13, but our identification of the EOMES-centered gene regulatory network provides unprecedented resolution into the transcriptional drivers of this disparity. The EOMES GRN, encompassing CD8A, GZMK, STAT5A, CXCR3, and LAG3, integrates cytotoxic effector functions, migratory capacity, and checkpoint regulation—features critical for sustained anti-tumor immunity. This GRN is significantly associated with CD8+ T cell activation, cytotoxicity, and proliferation. The heightened antigen-presenting capacity of mature B cells (B_cell_RFTN1) in females adds another layer to this immunological advantage. As B cell differentiation progresses, antigen presentation with CD8+ T cells intensify. In female CRC, differentiated B cells are more prevalent, whereas male CRC is dominated by immature B cells, which may potentially diminish the effectiveness of anti-tumor responses. In males, the accumulation of CAF_MMP11, a myofibroblastic subset marked by MMP11 and FN1 overexpression, reveals a stromal axis driving CRC progression. The THBS, MK, and FN1 signaling between CAF_MMP11 and epithelial cells further exacerbates tumor growth, as CAF-derived THBS2 and MK are implicated in therapy resistance and metastasis 46, 48, 49. The sex-specific TNF-TNFRSF1B axis between Macro_CCL4 macrophages and CD8+ T cells unveils a novel immune coordination mechanism. While TNF signaling is broadly implicated in T cell activation, its female-biased utilization underscores estrogen's role in fine-tuning myeloid-immune crosstalk. Macro_CCL4-derived TNF likely primes CD8+ T cells via TNFRSF1B, lowering TCR activation thresholds and amplifying cytotoxicity, corroborated by the strong correlation between TNF pathway activity and T cell effector scores.

## Conclusions

In summary, our study delineates sex-specific immunological and hormonal responses in CRC at the single-cell level, highlighting the significant impact of estrogen on the CRC tumor microenvironment and resulting in prognostic disparities between sexes. These insights advance our understanding for developing targeted CRC therapies.

## Supplementary Material

Supplementary figures and tables.

## Abbreviations

CRC: Colorectal cancer
TME: Tumor microenvironment
scRNA-seq: single-cell RNA sequencing
DPBS: Dulbecco's phosphate-buffered saline
DEGs: Differentially expressed genes
GO: Gene ontology
GSEA: Gene Set Enrichment Analysis
TCGA: The cancer genome atlas
DCs: Dendritic cells
PDCs: Plasmacytoid dendritic cells
CAFs: Cancer-associated fibroblasts
ECM: extracellular matrix
