# Supplementary material for: Notable influences of estrogen and sex-specific microenvironment in colorectal cancer revealed by single-cell transcriptome analysis
Source: Int J Med Sci. 2025 May 28;22(11):2637–52. doi: 10.7150/ijms.106133 (PMC12163387; doi:10.7150/ijms.106133)
Supplement: Supplementary file 1 — Supplementary figures and tables. [file ijmsv22p2637s1.pdf]

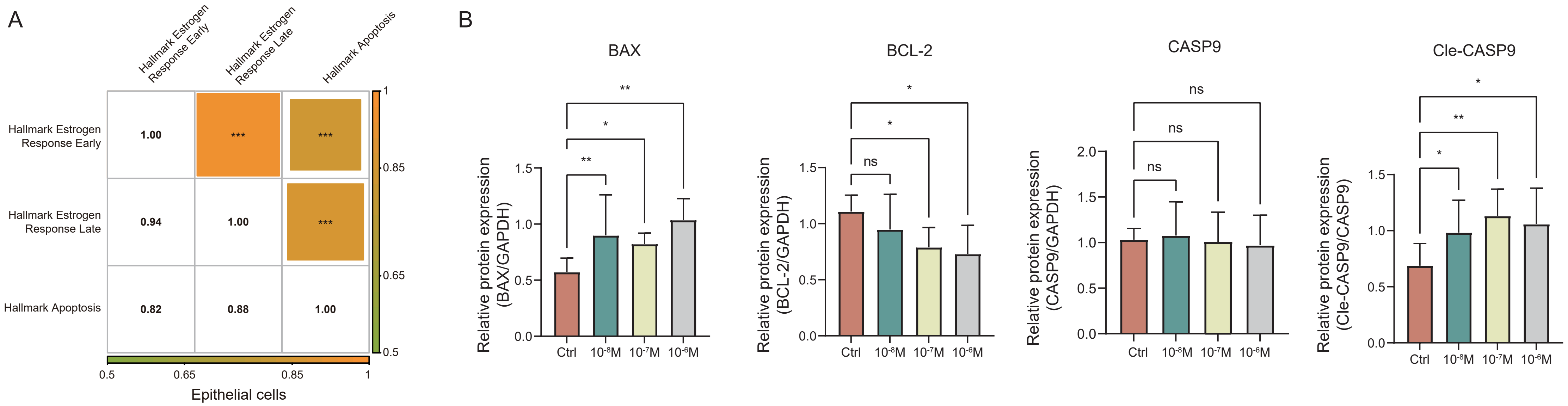

A
Hallmark Estrogen Response Early
Hallmark Estrogen Response Late
Hallmark Apoptosis
1.00
***
***
0.94
1.00
***
0.82
0.88
1.00
1
0.85
0.65
0.5
0.5
0.65
0.85
1
Epithelial cells
B
BAX
BCL-2
CASP9
Cle-CASP9
Relative protein expression (BAX/GAPDH)
Relative protein expression (BCL-2/GAPDH)
Relative protein expression (CASP9/GAPDH)
Relative protein expression (Cle-CASP9/CASP9)
Ctrl
10⁻⁸M
10⁻⁷M
10⁻⁶M
**
*
ns

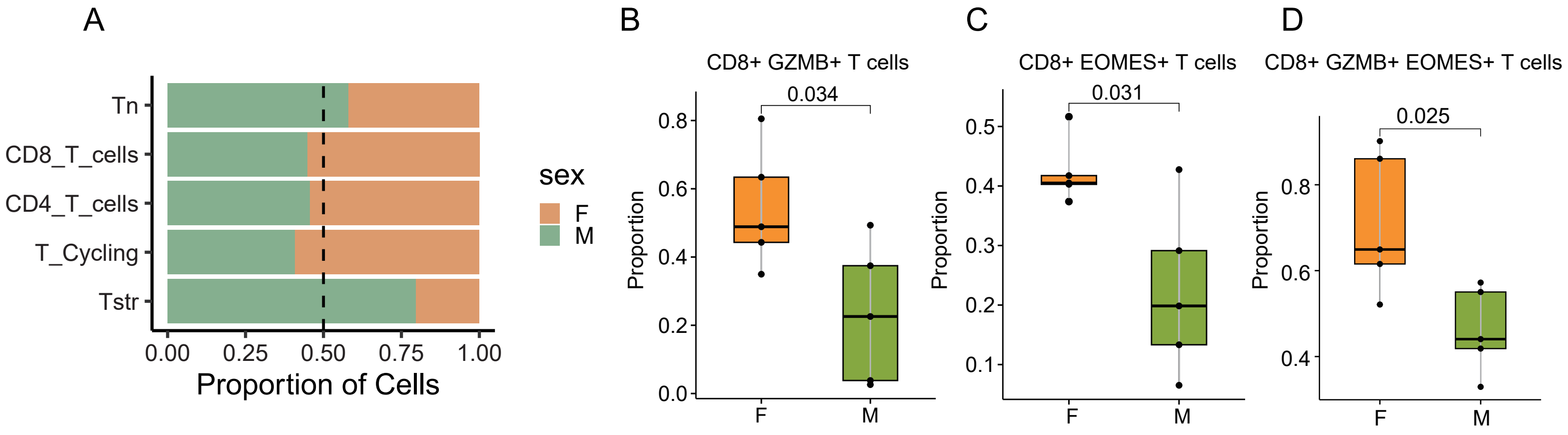
A
Tn
CD8_T_cells
CD4_T_cells
T_Cycling
Tstr
0.00
0.25
0.50
0.75
1.00
Proportion of Cells
sex
F
M
B
CD8+ GZMB+ T cells
0.034
Proportion
0.0
0.2
0.4
0.6
0.8
F
M
C
CD8+ EOMES+ T cells
0.031
Proportion
0.1
0.2
0.3
0.4
0.5
F
M
D
CD8+ GZMB+ EOMES+ T cells
0.025
Proportion
0.4
0.6
0.8
F
M

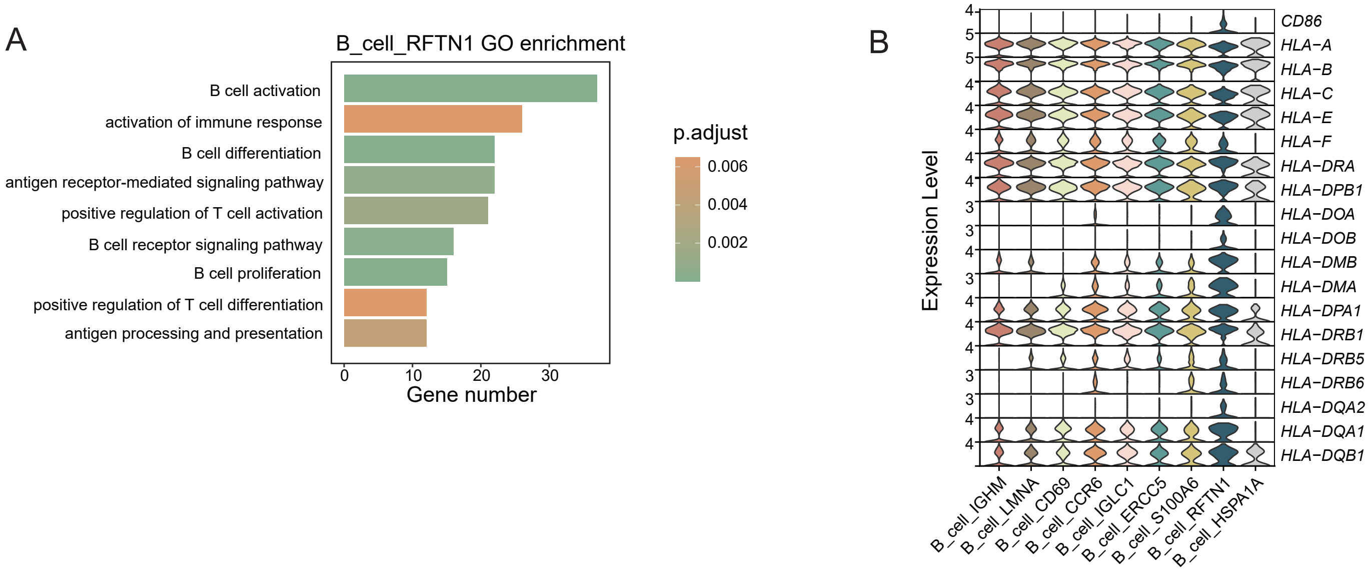
A
B_cell_RFTN1 GO enrichment
B cell activation
activation of immune response
B cell differentiation
antigen receptor-mediated signaling pathway
positive regulation of T cell activation
B cell receptor signaling pathway
B cell proliferation
positive regulation of T cell differentiation
antigen processing and presentation
0
10
20
30
Gene number
p.adjust
0.006
0.004
0.002
B
Expression Level
CD86
HLA−A
HLA−B
HLA−C
HLA−E
HLA−F
HLA−DRA
HLA−DPB1
HLA−DOA
HLA−DOB
HLA−DMB
HLA−DMA
HLA−DPA1
HLA−DRB1
HLA−DRB5
HLA−DRB6
HLA−DQA2
HLA−DQA1
HLA−DQB1
B_cell_IGHM
B_cell_LMNA
B_cell_CD69
B_cell_CCR6
B_cell_IGLC1
B_cell_ERCC5
B_cell_S100A6
B_cell_RFTN1
B_cell_HSPA1A

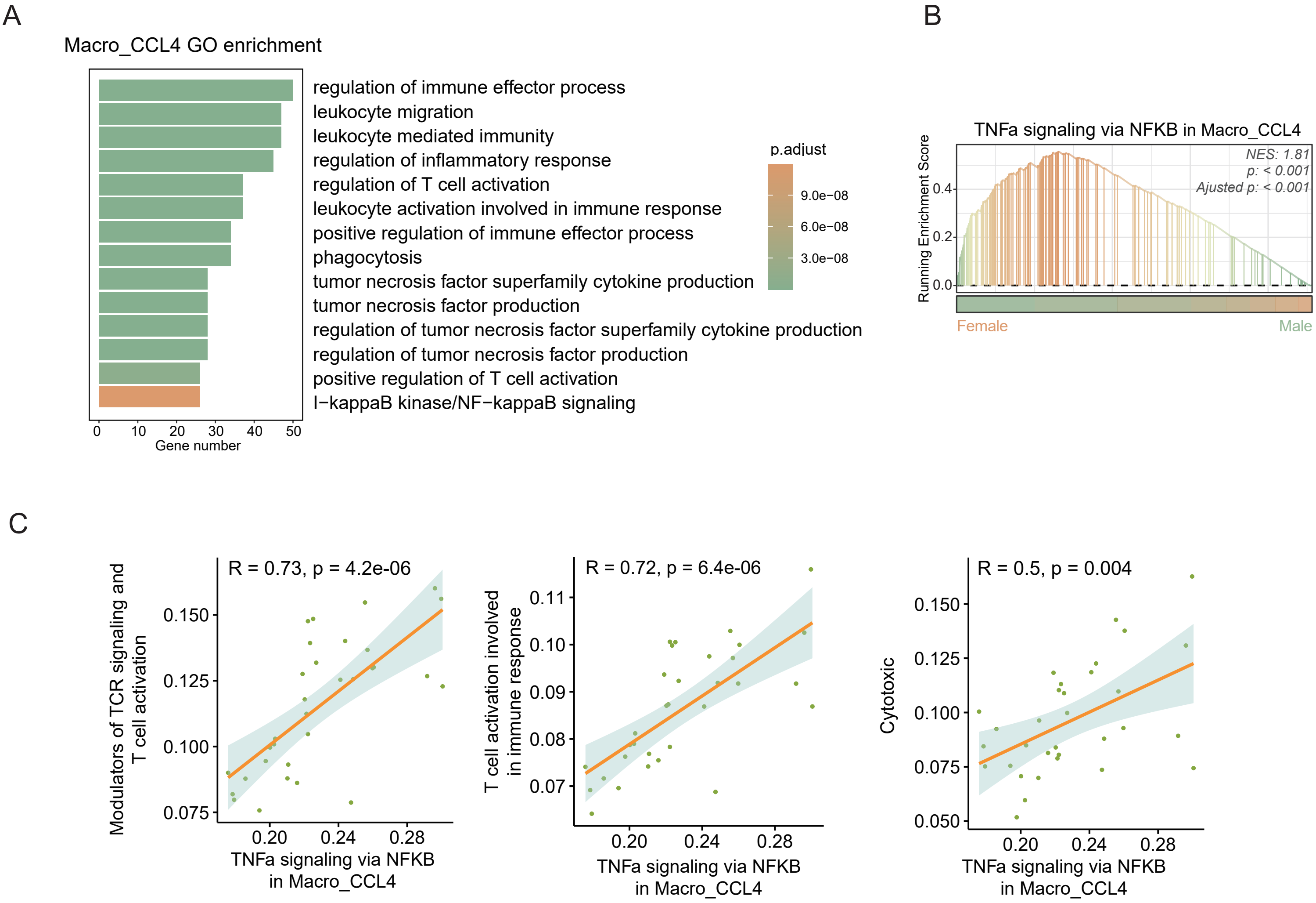

A
Macro_CCL4 GO enrichment
regulation of immune effector process
leukocyte migration
leukocyte mediated immunity
regulation of inflammatory response
regulation of T cell activation
leukocyte activation involved in immune response
positive regulation of immune effector process
phagocytosis
tumor necrosis factor superfamily cytokine production
tumor necrosis factor production
regulation of tumor necrosis factor superfamily cytokine production
regulation of tumor necrosis factor production
positive regulation of T cell activation
I−kappaB kinase/NF−kappaB signaling
0 10 20 30 40 50
Gene number
p.adjust
9.0e−08
6.0e−08
3.0e−08
B
TNFa signaling via NFKB in Macro_CCL4
Running Enrichment Score
0.4
0.2
0.0
NES: 1.81
p: < 0.001
Ajusted p: < 0.001
Female
Male
C
R = 0.73, p = 4.2e-06
Modulators of TCR signaling and T cell activation
0.150
0.125
0.100
0.075
0.20 0.24 0.28
TNFa signaling via NFKB in Macro_CCL4
R = 0.72, p = 6.4e-06
T cell activation involved in immune response
0.11
0.10
0.09
0.08
0.07
0.20 0.24 0.28
TNFa signaling via NFKB in Macro_CCL4
R = 0.5, p = 0.004
Cytotoxic
0.150
0.125
0.100
0.075
0.050
0.20 0.24 0.28
TNFa signaling via NFKB in Macro_CCL4

Fig. S1 Estrogen may promote apoptosis in cancer cells. A) Heatmap shows the correlation coefficients of gene set scores of Hallmark pathways in Epithelial cells. Significance levels are expressed as *p<0.05, **p<0.01 and ***p<0.001. Deeper color represents higher pearson correlation coefficient. **B)** Semi-quantification of the western blot analysis of protein expression levels of BAX, BCL-2, CASP9, and Cle-CASP9 in RKO and LS174T cell lines following treatment with varying concentrations of E2 (10^{-6} M, 10^{-7} M, 10^{-8} M) and control (Ctrl) conditions, n = 6. One-way ANOVA utilizing two-stage linear step-up procedure of Benjamini, Krieger and Yekutieli was used for statistical analysis. Significance levels are expressed as *p<0.05, **p<0.01 and ***p<0.001.

Fig. S2 The sex-specific differences in the EOMES transcription module affect the anti-tumor functions of CD8+ T cells. A) Bar plot shows the proportion of different sexes in the T cell subsets. **B)** Box plot shows the proportion of CD8+ GZMB+ T cells in CD8+ T cells. Each dot in the box plot represents one sample. t-test. **C)** Box plot shows the proportion of CD8+ EOMES+ T cells in CD8+ T cells. Each dot in the box plot represents one sample. t-test. **D)** Box plot shows the proportion of CD8+ GZMB+ EOMES+ T cells in CD8+ GZMB+ T cells. Each dot in the box plot represents one sample. t-test.

Fig.S3 Sex-biased functional differences in B cell antigen presentation. A) GO pathway enrichment of B_cell_RFTN1 comparing to other B cell subsets. **B)** Violin plot shows the expression of MHC-I and MHC-II genes among B cell subsets.

Fig.S4 Macrophages interact with CD8+ T cells via the TNF-TNFRSF1B ligand-receptor pair, showing higher intensity in female CRC. A) GO pathway enrichment of Macro_CCL4 comparing to other myeloid subsets. **B)** GSEA pathway enrichment of “TNFa signaling via NF-κB” between different sexes in Macro_CCL4. **C)** Correlation plots of TNFa Hallmark pathway score in Macro_CCL4 and gene set scores of pathways in CD8+ T cells.

Table S1. clinical characteristics of CRC patients in scRNA-seq cohort.

Patient.ID	Age	Sex	Diagnosis	Tumor stage	Histological type
CRC-F01	63	F	rectal adenocarcinoma	cT4aN2bM0	Moderately differentiated adenocarcinoma
CRC-F02	58	F	rectal adenocarcinoma	cT3bN1bM0	Moderately differentiated adenocarcinoma
CRC-F03	45	F	rectal adenocarcinoma	cT3bN0-1aM0	Moderately differentiated adenocarcinoma
CRC-F04	53	F	rectal adenocarcinoma	cT3aN2bM0	Moderately differentiated adenocarcinoma
CRC-F05	68	F	rectal adenocarcinoma	cT2-3aN0	intramucosal carcinoma
CRC-F06	36	F	rectal adenocarcinoma	cT3bN1bM0	Moderately differentiated adenocarcinoma
CRC-F07	35	F	rectal adenocarcinoma	cT3bN1bM0	Moderately differentiated adenocarcinoma
CRC-F08	70	F	rectal adenocarcinoma	cT4aN2M0-1	Moderately differentiated adenocarcinoma
CRC-F09	33	F	rectal adenocarcinoma	cT4bN1bM1	Moderately differentiated adenocarcinoma
CRC-F10	65	F	rectal adenocarcinoma	cT3bN0M0	Moderately differentiated adenocarcinoma
CRC-F11	59	F	rectal adenocarcinoma	cT4bN1bM1	Adenocarcinoma
CRC-F12	28	F	sigmoid colon adenocarcinoma	cT4aN2M0	Mucinous adenocarcinoma
CRC-F13	49	F	rectal adenocarcinoma	cT3bN2Mx	Adenocarcinoma
CRC-M01	52	M	rectal adenocarcinoma	cT3bN1bMx	Moderately differentiated adenocarcinoma
CRC-M02	22	M	rectal adenocarcinoma	cT3bN1aM1	Moderately differentiated adenocarcinoma
CRC-M03	22	M	rectal adenocarcinoma	cT3N1Mx	Mucinous adenocarcinoma
CRC-M04	36	M	rectal adenocarcinoma	cT3aN1-2M0	Moderately differentiated adenocarcinoma
CRC-M05	52	M	rectal adenocarcinoma	cT3cN2bM1	Moderately differentiated adenocarcinoma
CRC-M06	73	M	rectal adenocarcinoma	cT4aN2+Mx	Moderately differentiated adenocarcinoma
CRC-M07	68	M	rectal adenocarcinoma	cT4aN2bM1	Moderately differentiated adenocarcinoma
CRC-M08	74	M	rectal adenocarcinoma	cT3bN0Mx	Moderately differentiated adenocarcinoma
CRC-M09	44	M	rectal adenocarcinoma	cT3cN2M1b	Moderately differentiated adenocarcinoma
CRC-M10	67	M	rectal adenocarcinoma	cT4aN2bM1	Moderately differentiated adenocarcinoma

Table S1. clinical characteristics of CRC patients in scRNA-seq cohort.

CRC-M11	52	M	rectal adenocarcinoma	cT3bN0M0	Moderately differentiated adenocarcinoma
CRC-M12	49	M	rectal adenocarcinoma	cT4bN2M0	Moderately-poorly differentiated adenocarcinoma
CRC-M13	41	M	rectal adenocarcinoma	cT3cN2aM0	Mucinous adenocarcinoma
CRC-M14	64	M	rectal adenocarcinoma	cT4aN2bM0	Moderately differentiated adenocarcinoma
CRC-M15	60	M	rectal adenocarcinoma	cT4bN1Mx	Moderately differentiated adenocarcinoma
CRC-M16	43	M	rectal adenocarcinoma	cT4bN2bM0	intramucosal carcinoma
CRC-M17	49	M	sigmoid colon adenocarcinoma	cT3N2M0	Moderately differentiated adenocarcinoma
CRC-M18	65	M	rectal adenocarcinoma	cT3bN2M0	Moderately differentiated adenocarcinoma
CRC-M19	68	M	colon adenocarcinoma	cT3-4N1M0	Moderately differentiated adenocarcinoma

Table.S2 Gene list for epithelial scoring related to Fig.2d

Proliferation	Immune_surveillance	Immune_escape	Intestinal_stem_cell	Check_point
MCM5	HLA-A	CD47	AQP4	CD80
PCNA	HLA-B	ADAM10	OLFM4	CD86
TYMS	HLA-C	HLA-G	TNFRSF19	CD276
FEN1	MICA	CD274	CDCA7	VTCN1
MCM2	MICB	FASLG	PRELP	VSIR
MCM4		CCL5	RNF32	HHLA2
RRM1		TGFB1	RGMB	CD274
UNG		IL10	CLCA4	PDCD1LG2
GINS2		PTGER4	CDK6	BTN3A1
MCM6			ASCL2	TNFSF4
CDCA7			SOAT1	TNFSF9
DTL			SLC14A1	CD70
PRIM1			SCN2B	ICOSLG
UHRF1			LGR5	NECTIN2
MLF1IP				CD200
HELLS				CD48
RFC2				LGALS9
RPA2				TNFSF18
NASP				CD40
RAD51AP1				TNFRSF14
GMNN				PVR
WDR76				IDO1
SLBP				CTLA4
CCNE2				
UBR7				
POLD3				
MSH2				
ATAD2				
RAD51				
RRM2				
CDC45				
CDC6				
EXO1				
TIPIN				
DSCC1				
BLM				
CASP8AP2				
USP1				
CLSPN				
POLA1				
CHAF1B				

Table.S2 Gene list for epithelial scoring related to Fig.2d

BRIP1
E2F8
HMGB2
CDK1
NUSAP1
UBE2C
BIRC5
TPX2
TOP2A
NDC80
CKS2
NUF2
CKS1B
MKI67
TMPO
CENPF
TACC3
FAM64A
SMC4
CCNB2
CKAP2L
CKAP2
AURKB
BUB1
KIF11
ANP32E
TUBB4B
GTSE1
KIF20B
HJURP
CDCA3
HN1
CDC20
TTK
CDC25C
KIF2C
RANGAP1
NCAPD2
DLGAP5
CDCA2
CDCA8
ECT2

Table.S2 Gene list for epithelial scoring related to Fig.2d

KIF23
HMMR
AURKA
PSRC1
ANLN
LBR
CKAP5
CENPE
CTCF
NEK2
G2E3
GAS2L3
CBX5
CENPA

Materials and Methods

Human subjects

After obtaining approval from the Ethics Committee of the Sixth Affiliated Hospital of Sun Yat-sen University (No.2024ZSLYEC-240), this study enrolled 32 CRC patients diagnosed at the Sixth Affiliated Hospital of Sun Yat-sen University were enrolled in this study, including 19 males and 13 females. No statistically significant differences in age were observed between the sexes ($p = 0.7484$), tumor stage ($p = 0.8758$), or histological type ($p = 0.5427$). Detailed clinical and pathological information are presented in Table S1.

Single-cell suspensions, library construction, and sequencing

Fresh specimens of tumor tissues were carefully cleaned with Dulbecco's Phosphate-Buffered Saline first and then cut into 1-2 mm^3 cubes on ice, while kept on ice. Enzymatic digestion was conducted using the MACS Human Tumor Dissociation Kit (Miltenyi Biotec) on these tissue fragments. Single-cell 3'-libraries were prepared using the DNA Nanoball (DNB) elab C4 scRNA Preparation Kit in following the manufacturer's protocol. After library construction, sequencing was performed on the DNBelab C4 sequencing platform, and the raw reads were processed with stringent filtration and demultiplexing using the PISA software for accurate data analysis (https://github.com/shiquan/PISA).

Single-cell RNA-seq data processing

The refined sequencing reads aligned to the human genome using the STAR (v.2.7.4a), and sorted with Sambamba (v.0.7.0). The resulting cell-gene count matrix was then imported into Seurat R package (v.4.2.2) to create a Seurat object, ready for subsequent analysis [16].

To ensure the quality of our downstream analysis, we implemented stringent filtering criteria to select only high-quality cells. We excluded cells based on the following criteria: 1. Cells with more than 20% mitochondrial transcripts, indicating potential cell stress or damage. 2.Cells expressing fewer than 300 genes or more than 6,000 genes, which could represent low-quality or highly variable cells. 3.Doublets

and contaminant cells identified by disordered clustering in the UMAP embedding space or chaotic marker expression. 4.Cells with high read depths exceeding 30,000 UMI counts, which may indicate technical artifacts or over-amplification. After filtering, we normalized the discrete gene expression counts across individual cells within each sample using the "LogNormalize" function from Seurat. This normalization step helps to reduce the impact of gene expression variability due to differences in sequencing depth and allows for more accurate comparisons between cells. Next, we employed the "FindVariableFeatures" function from Seurat (v.4.2.2) to identify the top 3,000 genes with high variability, termed Highly Variable Genes. These HVGs are crucial for capturing the biological variability within the dataset and are essential for subsequent dimensionality reduction and clustering analyses. For UMAP projection and clustering analysis, we utilized the top 30 principal components and set a resolution of 0.4.

Cell type annotation

We identified various cell types, including B cells, plasma cells, epithelial cells, endothelial cells, myeloid cells, fibroblasts, pericytes, mast cells, T cells, subsets of T cells, and subsets of myeloid cells, using classic cell-type markers. Subsets of tumor cells, B cells, myeloid cells, and fibroblasts were further classified based on differentially expressed genes using Seurat's "FindAllMarkers" function.

Gene signature score

We employ the R package AUCell [17] (v1.20.2) to assess cellular functions and signaling pathways on the normalized matrix of Seurat objects. The gene lists for this analysis are provided in Table S2. We utilized the myeloid cell function gene list from Sun et al [18] in Figure 5D and the T-cell cytotoxic and proliferative score gene list from Huang et, al [19] in Figure 3C, G.

Pathway Enrichment analysis

Using the clusterProfiler [20] (v4.6.2) and GSEABase [21] (v1.60.0) R packages, we performed a comparative pathway enrichment analysis on the Seurat object to identify sex-specific differences in enriched pathways. Significance was ascertained with a stringent p cutoff of less than 0.05.

Cell-cell interaction analysis

Using the CellChat [22] (v1.6.1) R package, we conducted an intercellular communication analysis to explore the interactions between distinct cell types. Following the official workflow, we transformed Seurat objects into CellChat objects. We then calculated ligand-receptor pairs and their communication probabilities based on the CellChatDB human database. Ligands and receptors expressed in fewer than ten cells within a cell type were excluded from the analysis.

Transcription factor and gene regulatory network analysis

We utilized the SCENIC [17] (v1.1.2.2) R package to analyze the regulatory networks involving transcription factors (TFs) and their target genes. By quantifying gene regulatory networks (GRNs) at the single-cell level, we mapped the regulatory interactions between TFs and their targets. Each regulatory module was assigned AUC scores to assess their activity. The analysis aimed to identify TFs with significant regulatory impact across different cell types and sexes by evaluating the regulatory intensity of TFs and their target genes. This approach helps to uncover the key regulatory elements that may contribute to sex-specific differences in cellular behavior within the tumor microenvironment.

Developmental trajectory analysis

We employed Monocle2 [23] algorithm for cell trajectory analysis, utilizing the "DDRTree" method to perform dimensionality reduction and order cells based on differentially expressed genes specific to B cell subsets. Additionally, we applied the Cytotrace [24] algorithm to predict cell ordering, adhering to its official workflow for accurate trajectory inference.

Ro/e tissue preference analysis

We used a well-established approach from prior literature [25] to assess sex-based tissue preference by calculating the observed-to-expected ratios (Ro/e) for various cell types. A Ro/e ratio > 1 indicated enrichment of that cell type in a particular sex. "+++" stands for Ro/e score > 1, "++" stands for Ro/e score ≤ 1 but > 0.8. "+" stands for Ro/e score ≤ 0.8 & ≥ 0.2. "+/−" stands for Ro/e score ≤ 0.2 & > 0. "-" stands for Ro/e score = 0. This quantification helped us identify cell types with

significant sex-based differences in tissue distribution.

Survival analysis

To perform single-gene survival curve analysis, bulk RNA-seq data, including clinical information and gene expression matrices, were obtained from the Cancer Genome Atlas Program (https://www.cancer.gov/ccg/research/genome-sequencing/tcga). The optimal cutoff value for gene expression was determined using the "surv_cutpoint" function from the survminer (v0.4.9) package. Survival curves were then generated with the "survfit" function from the survival (v3.4-0) package and visualized using the ggsurvplot function.

Multi-color immunohistochemistry

We conducted multi-color immunohistochemistry on paraffin-embedded CRC sections collected from the Sixth Affiliated Hospital of Sun Yat-sen University. The cohort consisted of 5 male and 5 female CRC cases, none of which had undergone prior neoadjuvant therapy.

The paraffin sections were first heated in an oven at 65℃ for one hour, then deparaffinized in xylene, followed by rehydration through a graded series of solutions, including anhydrous ethanol, 95% ethanol, 75% ethanol, and distilled water. Staining was performed according to the manufacturer's protocol using the PANO 5-plex IHC Kit (Cat#10002100100, Panovue). Antigen retrieval was carried out by heating the sections in citrate solution (pH 9.5, ZSGB-BIO) in a pressure cooker for 18 minutes. Following this, the sections were incubated with blocking buffer at 37℃ for 10 minutes. Rabbit anti-human EOMES (Abcam, clone EPR21950-241, 1:50) was applied and incubated overnight at 4℃, followed by the addition of a secondary horseradish peroxidase-conjugated antibody (Panovue) and incubation at 37℃ for 10 minutes. Signal amplification was performed using the TSA working solution, diluted 1:100 in amplification diluent (Panovue), with a 10-minute incubation at 37℃. Subsequently, rabbit anti-human GZMB (CST, clone D6E9W, 1:200) and mouse anti-human CD8 (CST, clone C8/144B, 1:200) were incubated at 37℃ for 1 hour, with the subsequent steps following the same protocol. Finally, nuclei were stained

with DAPI. Image acquisition was performed using the TissueFAXS cytometry platform, and images were analyzed using StrataQuest software.

Cell culture and treatment

Human LS174T (#STCC10816, Servicebio) and human RKO CRC cell lines (#STCC00054P-1, Servicebio) were cultured in Roswell Park Memorial Institute-1640 medium (Corning) supplemented with 10% fetal bovine serum (Procell), and 1% penicillin and streptomycin (ThermoFisher) at 37°C in a humidified 5% CO_2 chamber. 1Mβ-estradiol (E2, #50-28-2, Merck) was dissolved in 1 mL of anhydrous ethanol by gentle rotation. Subsequently, 49 mL of sterile culture medium was added to the solution, and the mixture was serially diluted to final concentrations of 10^{-6} M, 10^{-7} M, and 10^{-8} M. After incubation with these estrogen concentrations or vehicle (2% ethanol) for 48 hours, cells were subjected to Western blot and TUNEL staining to assess the level of apoptosis.

Western blot

LS174T cells and RKO cell lines were each set up with control groups, E2-treated groups (10^{-6} M, 10^{-7} M, and 10^{-8} M). After incubation with E2 for 48 hours, proteins were extracted from the cells. The protein concentration of the samples was determined using a BCA assay kit (#G2026, Servicebio). Total proteins were then separated by 10% SDS-PAGE (Vazyme) and transferred onto PVDF membranes (#ISEQ00010, Millipore). The membranes were blocked with a rapid blocking solution (#G2052, Servicebio) and subsequently incubated overnight at 4°C with primary antibodies against BAX (#D2E11, 1:1000, Cell Signaling Technology), BCL-2 (#R22494, 1:1000, ZEN BIO), CASP9 (#R22844, 1:1000, ZEN BIO), Cleaved-CASP9 (Cle-CASP9, #R381336, 1:1000, ZEN BIO) and GAPDH (#ZB15004-HRP-100, 1:3000, Servicebio). The membranes were then incubated with HRP-conjugated goat anti-rabbit IgG (#GB23303, 1:10000, Servicebio). Finally, the proteins were visualized using ECL reagent (#G2020, Servicebio). Semi-quantitative analysis was performed using ImageJ software.

TUNEL staining

Cell apoptosis was assessed by TUNEL staining using the TMR (red) TUNEL

Cell Apoptosis Detection Kit (#G1502, Servicebio). RKO and LS174T cell lines were plated at a density of 1×10^5 cells per 35mm dish. Cells were fixed with 4% paraformaldehyde solution (dissolved in PBS) and permeabilized with Proteinase K for 10 minutes. Positive controls were prepared by treating samples with DNase I (#G3342, Servicebio). The TdT incubation buffer was prepared according to the manufacturer's instructions. After incubation in the dark for 1 hour, nuclei were stained with an anti-fade mounting medium containing DAPI (#G1407, Servicebio), and images were captured using a Pannoramic 250 FLASH III Digital Scanner.

Statistics and Reproducibility

We utilized unpaired two-tailed Wilcoxon rank-sum tests to assess differences in cell distribution between sexes. One-way ANOVA using the two-stage linear step-up procedure by Benjamini, Krieger, and Yekutieli was employed to determine inter-group differences during multiple comparisons. Pearson correlation coefficients were employed for correlation analysis to examine the relationships between genes and gene sets, as well as between different gene sets. $p < 0.05$ is considered statistically significant. All statistical analyses and data presentations were performed by the R program (v 4.2.2).
